# The Influence of Stimuli Valence and Arousal on Spatio-Temporal Representation of a Route

**DOI:** 10.3390/brainsci11060814

**Published:** 2021-06-19

**Authors:** Francesco Ruotolo, Filomena L. Sbordone, Ineke J. M. van der Ham

**Affiliations:** 1 CogScIVR, Department of Psychology, University of Campania “Luigi Vanvitelli”, 81100 Caserta, Italy; filomenaleonela.sbordone@unicampania.it; 2 Helmholtz Institute, Experimental Psychology, Utrecht University, 3584 CS Utrecht, The Netherlands; 3 Department of Health, Medical and Neuropsychology, Leiden University, 2333 AK Leiden, The Netherlands; c.j.m.van.der.ham@fsw.leidenuniv.nl

**Keywords:** route representation, spatial memory, arousal, valence, emotions

## Abstract

This study assesses the influence of valence and arousal of element/landmarks along a route on the spatio-temporal representation of the route itself. Participants watched a movie of a virtual route containing landmarks with high arousal and positive (HP) or negative valence (HN), or landmarks with low arousal and positive (LP) or negative valence (LN). Afterwards, they had to (a) imagine walking distances between landmarks, (b) indicate the position of the landmarks along the route, (c) judge the spatial and temporal length of the route, and (d) draw the route. Results showed that the tasks were differentially influenced by the valence and arousal levels. Specifically, participants were more accurate in representing distances between positive, rather than negative, landmarks and in localizing positive high arousing landmarks. Moreover, the high arousing landmarks improved performance at the route drawing task. Finally, participants in the negative and low arousing conditions judged the route as being metrically and temporally longer than participants in positive and high arousing conditions. These results are interpreted in the light of theories about the effects of emotions on memory processes and the “feelings-as-information” theory. In brief, the results support the idea that representations of a route reflect a combination of cognitive and emotional processes.

## 1. Introduction

While walking a route, people may come across environmental elements that may have emotional significance. For example, they can be attractive (e.g., a smiling baby) or frightening (e.g., an aggressive dog), relaxing (e.g., a verdant landscape), or sad (e.g., a barren cemetery). Such elements may be characterized by a specific valence, positive or negative, and they may induce a high (e.g., in case of fear or happiness) or low (e.g., in case of serenity or sadness) arousal level, that is, a different psychophysiological activation. Furthermore, these elements can represent reference points around which the mental representation of the route is built, just like environmental landmarks [1,2]. For this reason, studies suggest that the emotional salience of landmarks is a key dimension to consider when investigating how individuals represent environmental spatial information [3,4]. However, the influence of both valence and arousal of these elements on the mental representations of a route has hardly been explored [5]. Addressing this issue will deepen the scientific knowledge on how spatial memory works and, more generally, will allow for a better understanding of how people represent the spatiotemporal structure of everyday experiences.

An accurate representation of a route contemplates the integration of both spatial and temporal information, such as the direction of the route (e.g., right and left turns), its metric length, its temporal duration, as well as keeping track of the elements encountered during navigation, their position along the route and the distance at which they are placed from each other. The mental representation of all these aspects requires the cooperation of episodic memory and working memory processes [1,6,7,8,9,10,11]. Even more important for the current study is that the functioning of brain areas and cognitive processes involved in routes representation is modulated by emotions. An amygdala-hippocampus circuit reflects a relatively automatic effect of arousing elements on memory consolidation, whereas a prefrontal-hippocampus circuit is associated with a controlled encoding process and responds to the positive or negative valence of an element [12,13]; see also [14]. Therefore, these studies highlight the necessity to take into account both valence and arousal levels when exploring the way a route is mentally represented.

Several studies have shown that the valence and arousal levels of an element influence how its spatial and temporal features are encoded and retrieved. For example, it has been shown that participants have more difficulty retaining in working memory the location of multiple briefly presented highly-arousing items than the position of the non-arousing ones [15]. However, this effect is reversed when arousing items are encoded one at a time and the task involves long-term memory processes [16,17]. In this regard, Smith and colleagues [18] showed that an advantage for arousing over non-arousing elements emerged when the stimuli had a positive, rather than a negative, valence (see also [5,19]; but see [20,21] for an advantage of arousing negative stimuli). Similarly, Bergmann and colleagues [22] found that performance in a working memory task was accurate also with non-arousing stimuli but only when they had a positive valence. According to some authors, emotionally arousing elements would draw more attention and receive priority access to the working memory system as compared to the non-arousing ones [16,17,23,24]; see also [25,26]. Additionally, the presence of positive elements increases dopamine, which is an important underlying biological mechanism for executive control and working memory [27]. Finally, several studies have shown that negative experiences are usually remembered as having elapsed more slowly than positive experiences [28,29,30,31,32]. This effect is more pronounced with high than low arousing stimuli [33]. This would happen because arousing negative stimuli determine more complex and “loaded” memories to be retrieved [34,35]; see also [26].

In sum, most of the previous studies show that the encoding and retrieval of spatial and temporal information of high arousing positive elements would be more accurate than that of low arousing negative elements. However, it is not yet clear whether this also applies to the representation of routes. To our knowledge, the only study that has addressed this question is by Ruotolo and colleagues [2]. Participants were exposed to virtual routes characterized by images with positive, negative, or neutral valence. Results showed that the positive images facilitated the memory of their position along the route, as well as the memory of the distance between them. Instead, a negative route was perceived as longer than neutral or positive routes. The only advantage of negative over neutral elements appeared when participants were asked to draw the direction of the route. The authors concluded by attributing these effects to the kind of valence of the stimuli. However, since in Ruotolo et al.’s [2] study, positive and negative images had a similar arousal level but higher than that of the neutral images, it is also possible that the mentioned effects were due to the arousal level of the stimuli, and/or to a specific combination of arousal and valence.

Therefore, the aim of the current study is to verify if valence and arousal levels of elements encountered during a virtual route play an interacting or independent role on the way participants mentally represent the spatio-temporal features of the route itself. To this end, participants watched a short movie of a virtual walk along a route containing positive (HP) or negative (HN) high arousing elements or positive (LP) or negative (LN) low arousing elements. Afterwards, participants’ memories of the route were assessed. Specifically, relative distances between landmarks were measured by using an adapted version of the Mental Scanning Task (i.e., the Mental Walking Task [36,37,38,39]); the absolute position of the images-landmarks was measured by asking participants to mark an image’s position relative to the total route length; the configurational information (i.e., the direction of the route) and the spatial and temporal dimensions of the route were measured by asking participants to draw the route and to judge the route length in meters and seconds. These tasks were chosen because they allow different route characteristics to be measured, from memory for landmarks to configurational, spatial, and temporal properties. Moreover, these tasks have proven to be sensitive to the effects of emotional versus non-emotional landmarks (see [2]).

Based on the evidence that the arousing elements would be encoded and retrieved more accurately than the non-arousing ones, and given the beneficial effects of the positive valence on working memory processes, it is possible to hypothesize at least three scenarios:

1. If valence and arousal levels play an independent role, a main effect of the arousal level and/or a main effect of valence in all tasks is expected. In regard to arousal, participants in HP and HN conditions will be more accurate overall than participants in LP and LN conditions. In regard to the valence, participants in HP and LP conditions will be more accurate overall than participants in HN and LN conditions;
2. If valence and arousal levels play an interacting role, based on the several studies showing an advantage for positive high arousal conditions, it is plausible to hypothesize that participants in the HP condition will be more accurate in all tasks than participants in all other conditions;
3. A third possibility is that both interaction and main effects would emerge, depending on the task at hand. However, since we cannot make specific hypotheses about the differential effects of arousal and valence, results from this experiment will also inform about the different weight of these two dimensions in the different tasks.

The first two scenarios would support previous evidence about the effect of emotions on memory processes, while the third scenario represents the novelty of the current study.

## 2. Materials and Methods

### 2.1. Participants

The sample size was determined with the software G*power 3.1.9.2 [40]. With an α = 0.05 and a power (1 − β) = 0.80, results showed that two hundred participants were sufficient to detect an effect size (Cohen’s f) = 0.20. According to Cohen’s [41,42], two large replication studies of published findings (i.e., [43,44]) and meta-analyses [45,46,47]; for a review, see [48]) a Cohen’s f = 0.20 represents a reasonable estimate of a theoretically meaningful effect size. Post-hoc power analyses confirmed that with two hundred participants and observed Cohen’s fs ranging from 0.20 to 0.47, the median power of our tests was 0.95.

Participants, all university students, were recruited via flyers or word of mouth at Utrecht University (The Netherlands). All participants had normal or corrected-to-normal visual acuity, with no neurological or psychiatric disorder, age range: 18–40. The experimental conditions emerged from crossing valence (positive and negative) and arousal (high and low) in a 2 × 2 between-participants design. Fifty participants were assigned to each condition: HP (high arousal, positive valence; 20 males, 30 females), LP (low arousal, positive valence; 21 males and 29 females), HN (high arousal, negative valence; 16 males and 34 females), LN (low arousal, negative valence; 21 males and 29 females). The assignment of participants to each experimental condition had previously been established in a completely random manner (e.g., first participant, HP condition; second participant, LN condition; third participant, LP condition and so on....). Specific ANOVAs showed no differences between groups in age (F < 1), anxiety levels (F(3, 196) = 1.54, *p* = 0.21), participants’ mood (in all cases F < 1), and subjective orientation abilities (F(3, 196) = 1.09, *p* = 0.35) (see Table 1 for details). Recruitment and testing were in conformity with the requirements of the Ethical Committee of the Faculty of Social and Behavioral Sciences of Utrecht University and the 2013 Declaration of Helsinki. Informed consent was obtained from all participants. The ethical approval code is: FETC15-054-amendment. Being part of a larger study, the HP and HN conditions had been combined with a neutral condition (not present in the current study) and reported also in a previous work to answer a different research question through a different type of analysis (see [2]). 

### 2.2. Setting and Materials

#### 2.2.1. Virtual Route

**Figure 1:**
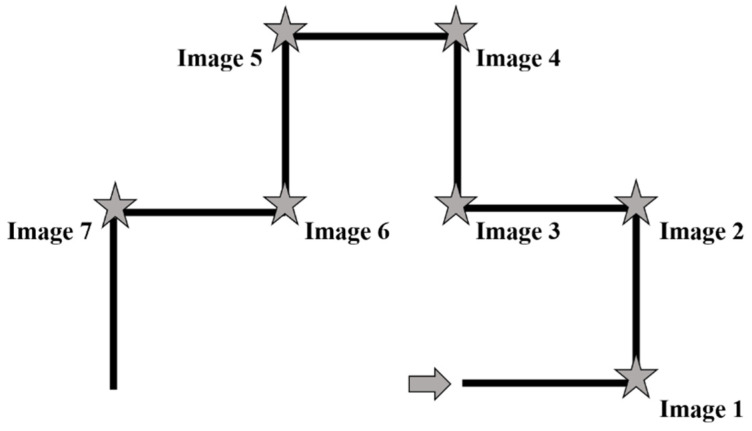
The figure depicts a 2D map of the route. The arrow indicates the direction and the starting point. The stars indicate the positions of the images along the route.

The virtual route was created by using the BLENDER open-source 3-D content creation suite. The virtual route was made up of eight hallways of the same length (the length was 5 Blender units for each segment, corresponding to about 35 meters of route length). There was an image/landmark centrally at the wall at the end of each hallway, except for the last one. The virtual route was the same for each condition except for the images. The exploration of the route was in first-person perspective and the viewpoint was set to 170 cm. The movie of the route was presented on a 32″ monitor by means of the software Open-Sesame and the speed of the exploration was at walking pace. A movie lasting 84 s showed the participant the entire route. See Figure 1.

#### 2.2.2. Images

Nineteen images were selected from the International Affective Picture System (IAPS Inventory, [51]) (see Table 2). We used images as landmarks and we interspersed emotionally laden images (4 images out of 7, that is, the first, third, fifth, and seventh) with neutral images (3 images out of 7, that is, the second, fourth, and sixth). The neutral images were the same for all the experimental conditions and were introduced for two reasons. First, to increase the ecological validity of the study (i.e., it’s not common to encounter a potential emotionally laden landmark at every corner of a route). Second, to measure the effect of each emotional landmark in the mental walking task and compare it across the experimental conditions. Indeed, participants were asked to mentally walk distances between neutral images and the emotional ones and vice-versa. For example, participants always started from the same neutral images (the same for all four experimental conditions) and imagined walking towards an emotionally laden image (which changed depending on the experimental condition) (see the procedure for further details on the mental walking task).

All the images were selected on the basis of a pilot study with 20 participants (all young university students). Since the IAPS does not contain the same images defined according to the four combinations of high/low arousal and high/low valence (for example, there is not the same image that has high valence and high arousal or low valence and high arousal etc.), the images have been selected according to the following criteria: (1) they were to be elements that a person usually meets or experiences during a walk (for this reason, images with a very low valence, such as “mutilated bodies” or scenes of “extreme violence”, and positive images with a very high valence, such as for example “pornographic images”, were excluded because they are also very uncommon to encounter along a route in daily life); (2) images with similar valence and arousal scores were to elicit the same emotion. For this reason, participants were presented with 200 images (50 for each combination of high/low valence and high/low arousal) and required to indicate what kind of emotion each image elicited. Only the images that received the agreement of 18 out of 20 participants were included. Results from this pilot study showed that HP images elicited happiness, LP images elicited serenity/calmness, HN images elicited fear, and LN images elicited sadness. As a result, HP images depicted a baby, puppies, a romantic scene, and a firework landscape. HN images depicted a snake, a dog, a gun, and a spider. LP images depicted sky with clouds, a flower, a garden, and a cornfield. LN images depicted rubbish, car smoke, shredded paper, and a snowy cemetery. Neutral images depicted common everyday objects. The selected images with the corresponding valence and arousal ratings for each condition are presented in Table 2.

Since the four experimental conditions contained different images, we verified whether the strength of the semantic relationship between the images of each experimental condition differed from the others. This is even more important due to the evidence that the strength of the semantic relationship between items influences the retrieval of temporal/episodic information of the same items (e.g., [52]): the stronger the semantic relationship, the more accurate the retrieval of information from memory. Therefore, we carried out a latent semantic analysis (LSA, [53]) on the conceptual contents of the images. In addition, we computed a further index of semantic similarity (ISS) using directly the images of each experimental condition. This was made with an ad-hoc questionnaire administered to 20 additional participants (see Appendix B for further details on both the analyses). Results from the Kruskal‒Wallis ANOVAs on both the indexes (i.e., LSA and ISS) revealed that the strength of semantic relationships among the landmarks did not differ across the experimental conditions (for the LSA: H(3) = 0.38, *p* = 0.94; for the ISS: H(3) = 4.76, *p* = 0.19).

Lastly, nineteen additional images were selected as distractors in the recognition task. These additional images were matched in terms of semantic contents, arousal, and valence with the images presented in the routes.

#### 2.2.3. Procedure

Before the experiment, participants completed the short-form of the State-Trait Anxiety Inventory (STAI, [49]), the Multidimensional Mood State Questionnaire [50], and a self-assessment question about their orientation abilities (0 = very bad–10 = excellent). Then, participants were seated in front of a 32” monitor. They were instructed to watch a movie of a virtual route and to memorize what they saw. After the movie, five tasks were presented in the same order for all participants: (1) recognition task, (2) mental walking task (MWT), (3) landmark position task (LPT), (4) spatial and temporal length judgment tasks (SLJT and TLJT), (5) route drawing task (RDT). When the movie stopped, the tasks were presented one at the time and their relative instructions were provided just before the starting of each task. After the experimental tasks, the Multidimensional Mood State Questionnaire was re-submitted to the participants to verify if and how participants’ mood had changed after the experiment. The experimental procedure lasted about 25 minutes in total.

*Recognition task*

This task assessed the ability to recognize the images previously seen in the route. Fourteen images were randomly presented to participants, seven presented in the route and seven distractors. Participants had to press the right button of a mouse if they recognized the image and the left button if not. This control task allowed us to be sure that all participants had seen and memorized the images along the route and that, above all, there were no significant differences between the four experimental conditions with regard to the recognition of the images.

*Mental walking task (MWT)*

The mental walking task is an adapted version of the mental scanning task [36,37,38,39]. The MWT measures the ability of the participants to represent the relative distances between landmarks within the virtual route. Indeed, it has been largely shown that this task informs of the relative distances between features of a learned configuration [54].

At first, participants were shown one image of the route (e.g., the fork) and were asked to form a visual image of the route and then to focus on the position of the image along the route (i.e., <<Try to visualize in your mind where this image was along the route>>). Once they had localized the image in their mind, they pressed the mouse button and two images appeared next to each other. The image on the left was that they were asked to mentally localize (i.e., the fork) and indicated the starting point of the mental walk for that trial. The image on the right (e.g., the aggressive dog) indicated the location to reach. Moreover, the image on the right was always located later along the route than the image on the left. Participants were asked to imagine walking from the left image to the right one and press the mouse button when they thought they had reached the position of the image on the right (i.e., <<Imagine walking from where you met the image on the left to where you met the image on the right. Press the space bar when you think you have reached the position of the right image>>). To investigate the impact of each emotionally laden landmark on the structural properties of the mental route, participants were asked to scan distances between neutral images and the emotional ones and vice-versa. Taking into account this constraint, we obtained three different distances: six short distances, four medium distances, and two long distances. Short distances were made up of two consecutives images (i.e., from image 1→2; 2→3; 3→4; 4→5; 5→6; 6→7), whereas medium and long distances had two (i.e., from image 1→4; 2→5; 3→6; 4→7) and four images (from image 1→6; 2→7) interpolated, respectively. A preliminary analysis was conducted to see if there was any difference from mentally going from an emotional image to a neutral one or from a neutral image to an emotional one, but no significant difference emerged (F < 1). So this distinction will no longer be considered.

*Landmark position task (LPT)*

In the LPT, all images from the route were presented one at a time in random order with a straight horizontal line below. Participants were instructed to consider the straight line as representing the total length of the route and to indicate where along the route the images was positioned. This task provided a continuous measure of geometric landmark properties. The metric error (in the units of the software used, i.e., virtual units) for each judgment was calculated by subtracting the indicated position from the actual position.

*Spatial (SLJT) and temporal (TLJT) length judgment tasks*

Participants were asked to indicate their estimation of the total length of the route in meters and the temporal duration of the route in seconds. The spatial and temporal errors in absolute values were calculated by subtracting the indicated lengths from the actual ones.

*Route drawing task (RDT)*

In the RDT, participants were asked to draw the virtual route by using straight lines on a sheet of paper (A4 size). The drawing reflected the accuracy of participants’ mental representation. The logic followed was to consider whether with that map in hand, another would be able to get to the end of the route correctly. Thus, if on the first turn the participant indicated right and not left, the map would have a zero score. Therefore, the accuracy was calculated as the sum of the correct consecutive turns along the route. The scores ranged from 0 to 7.

#### 2.2.4. Data Analysis

We analyzed participants’ performance for each task separately. Skewness and Kurtosis values for all the variables measured in this work are reported in Appendix A. For each task, outlier values were removed with Thompson’s tau technique [55]. Values removed were less than 5% of total data and the missing values, if any, were substituted with the average value of the condition.

The following analyses were carried out:

(i) First, we ensured that there were no differences between the four experimental conditions using a one-way ANOVA on accuracy at the recognition task;
(ii) Second, we checked through t-tests whether in each experimental condition the mood of the participants had changed by comparing the mood measured before and after the experiment. In this respect, we also measured the magnitude of mood change by subtracting the before mood from the after mood values for each of the mood dimensions considered (i.e., good‒bad, awake‒tired, calm‒nervous). The three dimensions of mood change were then used as covariates in the rest of the analyses. This was done to evaluate the effect of the various experimental conditions net of possible changes in participants’ mood;
(iii) Third, 2 × 2 ANCOVAs were performed on the other five tasks (i.e., MWT, LPT, SLJT, TLJT, RDT) separately to verify the presence of a main effect of valence (positive vs. negative), arousal (high vs. low), and/or an interaction between the two factors whilst controlling for the changes in mood. A Tukey HSD test was used to analyze post-hoc effects. Importantly, in no case did the covariates interact with the two main factors (i.e., valence and arousal levels). Moreover, to adjust for multiple testing, the false discovery rate “Benjamini and Hochberg” (FDR/B–H, [56]) procedure was used. The adjusted critical p-values, henceforth B–H values, were used to assess the statistical significance of each test. Only the effects that survived correction for multiple comparisons are reported and discussed;
(iv) In order to better understand the results observed with the ANCOVAs, a correlation analysis (Pearson) was carried out to explore what aspects might overlap across the different tasks.

## 3. Results

(i) *Recognition task.* In this task the recognition average accuracy (range 0–1) was the dependent variable. Results showed a high level of accuracy for all the conditions (mean accuracy range 0.92–0.95) and no significant differences appeared from the one-way ANOVA (F(3, 196) = 1.97, *p* = 0.12, ƞ^2^_p_ = 0.02).
(ii) *Mood changes of participants.* To verify if and in what way the mood reported by participants had changed after the experiment, we compared the scores for the three mood dimensions before and after the experiment (i.e., good–bad, awake–tired, calm–nervous) (see Table 3). Participants in the HP conditions reported the same good mood as before the experiment. Instead, participants in the LP condition reported feeling worse and more tired than before, but their level of calmness remained quite the same. Instead, participants in the HN condition reported being more nervous and worse than before but the level of awakeness remained the same as before. Finally, participants in the LN condition reported a worsening in all the mood dimensions.

Since the mood of the participants had changed in different ways according to the experimental condition, the values of the before mood were subtracted to the after mood for each of the mood dimensions. The three mood changes (MC) (i.e., MC good‒bad; MC awake‒tired; MC calm‒nervous) were used as covariates in the analyses of MWT, LPT, SLJT, TLJT and RDT.

(iii) *Spatio-Temporal Tasks:*

**Figure 2:**
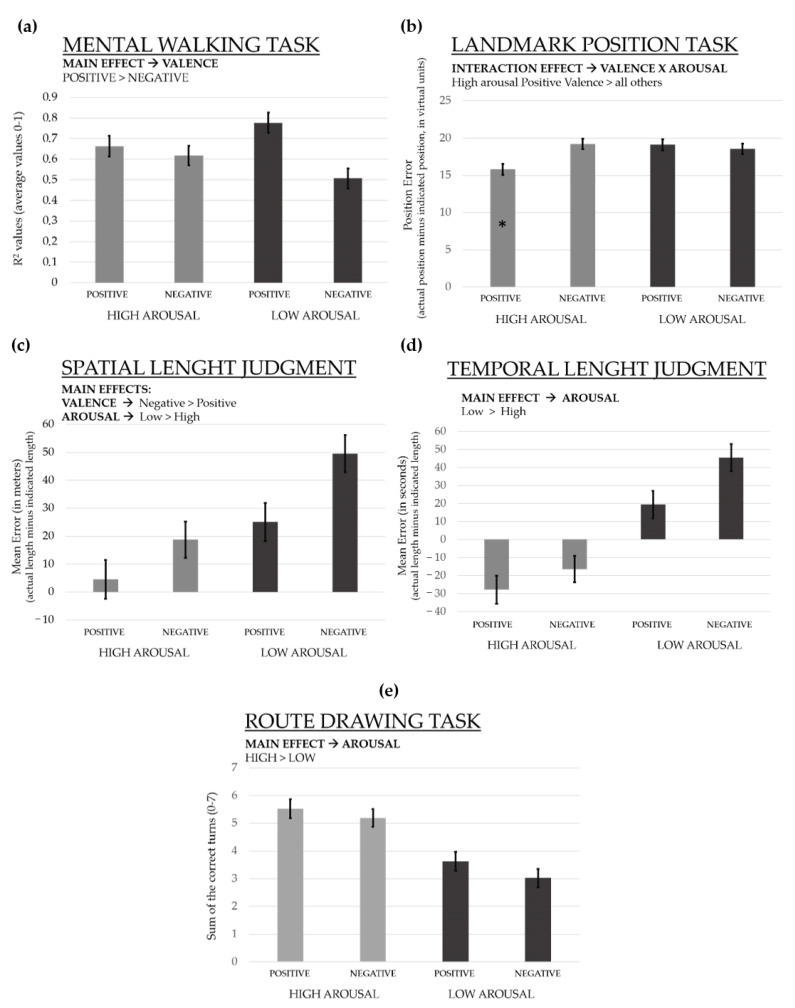
The graphs represent participants’ performance in the five tasks (**a**–**e**) as a function of the experimental condition (high or low arousal and positive or negative valence). The vertical black thin bars represent standard error. * *p* < 0.05.

*Mental walking task (MWT).* Standardized regression coefficients (adjusted R squared) have been used as a measure of accuracy. If the distances between the images had been accurately represented, then as the distance between two images increases the time taken by the participants to mentally cover that distance should increase. R2 values express the strength of this linear relationship. In other words, the higher was the R2 value, the more accurate was the performance. Adjusted R2 were calculated for each participant by using the three lengths as predictor variable (i.e., 0, 2, and 4 images interpolated between the two images) and participants’ response times for short, medium, and long distances as criterion variable. Results from the 2 × 2 ANCOVA showed a main effect of valence: F(1, 193) = 11.16, *p* = 0.0009 (B-H = 0.008), ƞ^2^_p_ = 0.05 (Cohen’s f = 0.24). Participants in positive conditions were more accurate (M = 0.72, SE = 0.03; 95% CI: 0.65–0.78) than participants in negative conditions (M = 0.56, SD = 0.03; 95% CI: 0.50–0.63). No other significant effect of either the main factors or the covariates appeared; see Figure 2a.

*Landmark position task (LPT).* The dependent variable was the position error (i.e., the average of the differences between the indicated and actual position of the seven images). Results of the 2 × 2 factorial ANCOVA revealed an interaction effect between valence and arousal: F(1, 193) = 8.38, *p* = 0.001 (B-H = 0.01), ƞ^2^_p_ = 0.04 (Cohen’s f = 0.21). The post-hoc analysis (Tukey HSD test) showed that the difference was due to participants in HP (M = 15.82, SE = 0.73; 95% CI: 14.37–17.27) condition being more accurate than participants in all other conditions (at least *p* < 0.05) (LP M = 19.09, SE = 0.73, 95% CI: 17.65–20.54; HN M = 19.23, SE = 0.63, 95% CI: 17.86–20.59; LN M = 18.55, SE = 0.71, 95% CI: 17.14–19.95); see Figure 2b. No other significant effect of either the main factors or the covariates appeared.

*Spatial (SJLT) and temporal (TJLT) length judgments tasks.* The dependent variable was the absolute error (i.e., the difference in absolute terms between the actual spatial and temporal length of the route and that indicated by the participants). For SJLT, results from the 2 × 2 ANOVA showed a main effect of arousal: F(1, 193) = 13.48, *p* = 0.0003 (B-H = 0.007), ƞ^2^_p_ = 0.06 (Cohen’s f = 0.26). The effect was due to participants in the low arousal condition being less accurate (M = 44.32, SE = 4.01; 95% CI: 36.40–52.23) than participants in the high arousal conditions (M = 21.47, SE = 4.01; 95% CI: 13.55–29.38). A main effect of valence also appeared: F(1, 193) = 16.68, *p* = 0.00006 (B-H = 0.005), ƞ^2^_p_ = 0.08 (Cohen’s f = 0.29). The effect was due to the participants in the negative conditions being less accurate (M = 43.48, SE = 3.62; 95% CI: 36.33–50.63) than participants in the positive conditions (M = 22.30, SE = 3.62; 95% CI: 15.15–29.45) (see Figure 2c). The descriptive statistics on length judgments reported by the participants showed that the actual route spatial length (i.e., 35 m) was overestimated more in the low (M = 72.34 m) than high arousal (M = 50.68 m) conditions, and more in the negative (M = 75.36 m) than positive (M = 47.65 m) conditions. No other significant effect of either the main factors or the covariates appeared.

As regard the TLJT, results showed a main effect of the covariate good‒bad mood change: F(1, 192) = 7.74, *p* = 0.006 (B-H = 0.01), ƞ^2^_p_ = 0.04 (Cohen’s f = 0.20). A main effect of arousal also appeared: F(1, 192) = 43.13, *p* = 0.000001 (B-H = 0.002), ƞ^2^_p_ = 0.18 (Cohen’s f = 0.47). Participants in the low arousal conditions were less accurate (M = 60.09, SE = 3.92; 95% CI: 52.36–67.82) than participants in the high arousal conditions (M = 20.23, SE = 3.90; 95% CI: 12.53–27.93) (see Figure 2d). The descriptive statistics on length judgments showed that the actual route temporal length (i.e., 84 s) was overestimated in low arousal conditions (M = 116.45 s) and underestimated in high arousal conditions (M = 61.90 s). Finally, to better understand the role of the covariate, a Pearson correlation analysis was carried out between the good‒bad mood change and the absolute error at the TLJT. Results tended to show that as mood change increased, the error of the temporal length estimation increased in the LP (*r* = 0.25, *p* = 0.08), HN (*r* = 0.22, *p* = 0.12), and LN (*r* = 0.29, *p* = 0.05) conditions. In other words, an increased negative mood was associated with an increased perceived temporal route length.

*Route drawing task (RDT)*. The sum of the correct consecutive turns along the route indicated by participants (range 0–7) was the dependent variable. Results from the ANOVA revealed only a main effect of arousal: F(1, 193) = 28.82, *p* = 0.000001 (B-H = 0.003), ƞ^2^_p_ = 0.13 (Cohen’s f = 0.39). The effect was due to participants in the high arousal being more accurate (M = 5.36; SE = 0.24; 95% CI: 4.88–5.84) than participants in the low arousal condition (M = 3.32; SE = 0.24; 95% CI: 2.84–3.80); see Figure 2e. No other significant effect of either the main factors or the covariates appeared.

(iv) *Correlational analysis*

A Pearson product-moment correlation was run to determine the relationship between the five tasks. All the results are presented in Table 4. Results showed that the spatial length judgments (SLJT) did not significantly correlate with temporal length judgements (TLJT). Instead, performance at the mental walking (MWT) significantly correlated with performance at the landmark position task (LPT): the higher the accuracy at MWT, the higher the accuracy at LPT. Instead, participants’ performance in the route drawing task (RDT) significantly correlated more with temporal (*r* = −0.32) than with spatial (*r* = −16) length judgments task, specifically, the higher the accuracy at RDT, the higher the accuracy at the TLJK and, to a less extent, at the SLJT.

## 4. Discussion

How do the arousal levels and valence of stimuli along a route influence the way individuals represent the spatio-temporal characteristics of the route itself? This study answered this question by asking four groups of participants to watch a movie of a virtual route containing images with a positive or negative valence and a higher or lower arousal level and by asking them to perform some spatial and temporal judgments. Overall, results supported the hypothesis that the arousal and valence of the landmarks may have either an independent or interacting role according to the task at hand. Before discussing the above mentioned effects, it is important to note that differences between the conditions cannot be attributed to a difficulty in remembering the correct images/landmarks or to a difference in mood or orientation abilities of the groups of participants. Highly accurate landmark recognition and mood and orientation ability were highly comparable across the different groups of participants. In addition, because participants’ mood at the end of the experiment had changed differently depending on the experimental condition, we used the mood change as a covariate in the main analyses. Overall, results showed no significant effect of mood change on participants’ performance on the different tasks. This indicates that the observed effects reflect the emotional content of the landmarks per se rather than the mood changes reported by participants after the experiment.

*Accuracy/Error rate*. Results showed that participants’ performance in the mental walking task, landmark position task, and spatial length judgments task was influenced by the valence of the stimuli. Specifically, participants in the positive conditions were more accurate than participants in the negative conditions. In contrast, participants’ performance in the route drawing task and in the temporal length judgments tasks were more influenced by the arousal level of the stimuli. Specifically, participants in the high arousal conditions were more accurate than participants in the low arousal condition.

In regard to the effect of *valence*, several studies have shown that the presence of positive items/elements improves working memory (e.g., for reviews [57,58]). The mechanisms by which this improvement occurs are not yet clear. Ashby, Isen, and Turken [27] suggest that the presence of positive items in HP and LP conditions may have induced an increase in dopamine, which is an important underlying biological mechanism for executive control and working memory. Instead, the presence of negative elements may have engaged attentional processes, and regulatory effort may have been activated, both consuming resources not more available for task execution ([59,60,61,62,63]; but for an advantage of negative elements on some aspects of spatial memory processes, please see [14,20,64]).

For *arousal*, several studies have shown that attentional processes are more influenced by individuals’ level of activation rather than by the valence of the arousing item. It has been suggested that the presence of a high arousing element catches the attention, thus producing a better recall of that element rather than of the contextual information (for a review, see [65]). According to Palombo and Cocquyt [26], an arousing item provides an emotional context allowing that item to be preferentially ‘encoded’ and strongly bound to its spatio-temporal dimension as compared to a non-arousing one. In turn, this stronger binding gives the arousing item an advantage also at ‘retrieval’ stage [25]. This would explain the advantage on some tasks of participants in high arousing conditions with respect to participants in low arousing conditions. Furthermore, it is important to clarify the difference between the current study and studies showing a destructive effect of arousing items on spatial working memory processes. For example, Mather and colleagues [15] found that participants remembered the spatial locations of multiple arousing elements presented briefly (<1 s) worse than the spatial location of non-arousing elements. Instead, in the current study, the arousing images/elements were presented one at a time along a route, for a much longer period (about 10 s per image), and alternated with neutral stimuli. Presumably, this procedure led to a deeper encoding phase, thus producing an advantage in retrieving the spatial information of highly-arousing elements (for similar results, see [16,17]).

Taken together, theories about the effects of emotionally laden items on memory processes would explain the advantages for positive and high arousing stimuli over negative and low arousing stimuli regarding the way the spatio-temporal features of a route are represented. However, why some tasks are more affected by arousal, such as the route drawing and the temporal length judgment task, and others by valence, such as the mental walking and the landmark position task, is less clear. The explanation probably lies in the role that temporal and spatial processes play in the different tasks and how these processes are differently influenced by arousal levels and stimulus valence. This would also be supported by the results of the correlations showing significant associations between performance on the route drawing and the temporal length judgment task on the one hand and between the mental walking and the landmark position task on the other. Future studies could help clarify this issue by exploring the role of temporal and spatial dimensions in the tasks used in the current study and verifying whether these dimensions are differently affected by the valence and arousal levels of the stimuli.

*Direction of spatial and temporal distortions.* Distortions in the spatio-temporal representation of a route can be easily observed in spatial and temporal length judgments tasks. Participants exposed to negative images judged the route as longer in both spatial and temporal terms as compared to those exposed to the positive images. The same happened in the case of routes with stimuli with low arousal levels as compared to those with high arousal levels. For the interpretation of these effects, we refer to the “feelings-as-information approach” by Schwarz and Clore [66,67]. This approach supports the idea that feelings provide a kind of information that is also motivating. For example, feeling scared has a negative effect on motivation for enterprise or adventure, whereas feeling energetic, optimistic, or happy may lead one out of the safety of one’s home and into the world. Importantly, the regulation of the behavior is exercised by influencing perceptions of the spatial layout. As a consequence, when we are tired or frightened, that is, we have low energy or we think it is not enough, people could tend to overestimate distances compared to when they are happy or in a positive mood. This is similar to what we found. Participants exposed to negative and low arousing images tended to judge distances as being longer than participants exposed to positive and high arousing stimuli. Furthermore, this is also in line with what was found by Blaison and Hess [68]. In their study, participants tended to overestimate the spatial extension of places with a negative salience (i.e., places characterized by “gang activity and drug traffic”) as compared to those with a positive salience. According to the authors, this biased appraisal could be due to an implicit avoidance strategy reflecting the desire to stay as far away as possible from negative places.

Similar results to those observed for spatial judgments were observed for temporal judgments. In fact, participants judged as temporally longer routes characterized by negative rather than positive images. Interestingly, the correlation analyses showed that an increase in perceived temporal route length was associated with an increase in participants’ negative mood after the experiment. Although these associations were not statistically significant (only trends were observed), they are in line with several studies showing that negative experiences are usually remembered as having elapsed more slowly than positive experiences [28,29,30,31,32]. However, results also showed that the effect of negative images was particularly strong when they were characterized by a low arousal level. This result is in contrast with some studies that show that the duration of high arousing stimuli is overestimated with respect to that of low arousing stimuli (e.g., [69]; see also [26]). According to some authors, the effect of emotions on temporal estimation cannot be reduced to the levels of arousal only, but it rather might depend on the meaning that a specific emotion has for individuals in a specific context [70,71]. For example, a disgusting event with a similar or lower arousal level of a scary one might be perceived as temporally longer because it would prompt more rapid activation of a defensive/avoiding strategy. Therefore, it is possible that the temporal overestimation we observed for the two low arousing conditions could be due to the kind of images used in the experiment.

*Limitations.* A limitation of this study concerns the fact that there was no measure of participants’ psychophysiological reactions during the routes. Furthermore, the mood questionnaire was administered after the participants had finished the entire experiment and not after the virtual path. This makes the interpretation of the results obtained in the LP condition less clear than in the other conditions. Indeed, participants in the LP condition showed a negative shift of the mood after the experiment as compared to participants in the condition with high arousing positive images (HP). Although their level of calmness remained the same, participants in the LP condition reported feeling less well and more tired than before the experiment. This negative shift could be due to the fact that the mood change might reflect not only the effect of the landmarks but also the difficulties in performing the tasks. In fact, participants in the LP condition had difficulties in performing some tasks, whereas participants in the HP condition did quite well in all the tasks. However, these speculations need further study to be addressed.

Another limitation is that the results cannot be generalized to all possible situations that we experience in our daily lives. For example, it sometimes happens that retracing the same route seems shorter even when we are tired [72]. For this reason, it is important to replicate this study in more ecological contexts and under different conditions of daily life. This is even more important in light of the fact that the source from which spatial information is learned (e.g., virtual scenarios vs. actual exploration of the environment) may influence how the spatial information is represented [73].

Finally, in the present study only tasks requiring an egocentric (i.e., based on the body and participants’ perspective) rather than an allocentric strategy (i.e., based on the relationship between environmental landmarks) were used [74,75]. As a consequence, we cannot generalize these results to the way individuals represent environmental knowledge like a map. Therefore, future studies are needed to verify if results found in this research can be generalized to more complex spatial configurations (e.g., routes with crossroads) and tasks (e.g., drawing maps by specifying at the same time both directions and landmarks; see [76]).

## 5. Conclusions

In conclusion, results from this study support the idea that the mental representation of a route reflects the interwoven effect of motivational, attentional, visuo-perceptual, and mental imagery mechanisms, which in turn involve working memory and long-term memory processes [7,77,78,79,80,81,82,83]. Importantly, all these mechanisms are differently influenced by the valence and arousal levels of the stimuli we might encounter along a route.

## Figures and Tables

**Table 1:** The table shows means and standard deviations (SD) for age, anxiety levels, mood, and perceived orientation ability for each experimental condition (i.e., HP, HN, LP, LN). N = number of participants; M = males; F = females.

	HP (N: M = 20, F = 30)		HN (N: M = 16, F = 34)		LP (N: M = 21, F = 29)		LN (N: M = 21, F = 29)	
	*Mean*	*SD*	*Mean*	*SD*	*Mean*	*SD*	*Mean*	*SD*
**Age**	20.82	3.85	21.08	3.98	21.78	4.26	20.88	2.91
**Anxiety ^a^**	9.28	2.15	9.28	2.86	10.22	2.90	10.06	3.38
**Mood ^b^ Good-Bad**	46.82	4.66	46.08	5.07	45.72	6.05	45.86	6.18
**Mood ^c^ Awake-Tired**	40.60	7.52	39.38	8.41	39.76	7.54	39.36	7.30
**Mood ^d^ Calm-Nervous**	44.52	5.43	44.70	6.17	43.88	8.52	44.18	7.42
**Orientation ^e^**	6.56	1.83	6.08	1.78	6.68	1.63	6.30	2.01

^a^ Anxiety levels were measured by means of the short version of the STAI (6 items; [49]) (scoring range: 6–24). ^b–d^ Participants’ mood was measured by means of the Multidimensional Mood State Questionnaire [50]. The dimensions measured are: good–bad, awake–tired, and calm–nervous (scoring range: 10 (very bad, very tired, very nervous)–60 (very good, very awake, and very calm)). ^e^ Participants were required to indicate on a 10-point grading scale how good they were able to orient themselves in the environment (0 = very bad; 10 = excellent).

**Table 2:** Images chosen for the experiment. The table reports the images chosen for the HP (high arousal positive valence), HN (high arousal negative valence), LP (low arousal positive valence), and LN (low arousal negative valence). Arousal and valence values for each image are those provided in the IAPS Inventory [51]. Each image is specified by the IAPS code in parentheses.

	HP		HN		LP		LN	
	Arousal	Valence	Arousal	Valence	Arousal	Valence	Arousal	Valence
1st Image	Smiling baby (2045)		Aggressive dog (1301)		Clouds (5891)		Garbage (9291)	
	5.47	7.87	6.02	4.26	3.29	7.22	4.38	2.93
2nd Image	Fork (7080)		Fork (7080)		Fork (7080)		Fork (7080)	
	1.98	5.43	1.98	5.43	1.98	5.43	1.98	5.43
3rd Image	Puppies (1710)		Snake (1022)		Flower (5010)		Exhaust gas (9090)	
	5.41	8.34	5.57	3.47	3.00	7.14	3.97	3.56
4th Image	Wire cutters (7056)		Wire cutters (7056)		Wire cutters (7056)		Wire cutters (7056)	
	3.24	4.98	3.24	4.98	3.24	4.98	3.24	4.98
5th Image	Kiss scene (4597)		Spider (1220)		Courtyard (5779)		Cemetery (9001)	
	5.91	6.95	5.77	3.7	3.57	7.33	3.67	3.10
6th Image	Plate (7233)		Plate (7233)		Plate (7233)		Plate (7233)	
	2.51	5.01	2.51	5.01	2.51	5.01	2.51	5.01
7th Image	Fireworks (5480)		Gun (6190)		Field (5725)		Shredded paper (7023)	
	5.48	7.53	4.83	4.52	3.55	7.09	4.17	3.80
Mean values ^a^	**Arousal**	**Valence**	**Arousal**	**Valence**	**Arousal**	**Valence**	**Arousal**	**Valence**
	5.54	7.77	5.55	3.93	3.35	7.20	4.04	3.35

^a^ Means of images 1, 3, 5, 7.

**Table 3:** Mean and standard deviation (SD, in parenthesis below the mean values) values for each mood dimension before and after the experiment. HP (high arousal positive valence), HN (high arousal negative valence), LP (low arousal positive valence), and LN (low arousal negative valence). Score range: 10 (very bad, very tired, very nervous)–60 (very good, very awake, very calm). Significant differences are indicated with * *p* < 0.05; ** *p* < 0.005; *** *p* < 0.0005.

	GOOD-BAD		AWAKE-TIRED		CALM-NERVOUS	
	**Before**	**After**	**Before**	**After**	**Before**	**After**
	Mean	Mean	Mean	Mean	Mean	Mean
	(SD)	(SD)	(SD)	(SD)	(SD)	(SD)
**HP**	46.82	46.02	40.60	40.34	44.52	43.40
	(4.66)	(5.38)	(7.11)	(7.66)	(5.43)	(7.14)
**HN**	46.08	42.96	39.38	39.24	44.70	41.94
	(5.06)	(4.29) **	(8.41)	(7.48)	(6.17)	(6.63) *
**LP**	45.72	35.72	39.76	36.74	43.88	42.16
	(6.05)	(6.75) ***	(7.54)	(7.47) *	(8.52)	(8.49)
**LN**	45.86	35.70	39.36	35.26	44.18	37.26
	(6.19)	(8.09) ***	(7.29)	(8.87) *	(7.42)	(10.39) ***

**Table 4:** The table reports the Pearson product-moment correlation coefficients (r) between the different tasks. Statistically significant r values are in bold. MWT = mental walking task; LPT = landmark position task; SLJT = spatial length judgment task; TLJT = temporal length judgment task; RDT = route drawing task.

	LPT (R, PVALUE)	RDT (R, PVALUE)	SLJT (R, PVALUE)	TLJT (R, PVALUE)
**MWT**	***r* = −0.16, *p* = 0.02**	*r* = −0.001, *p* = 0.89	*r* = −0.13, *p* = 0.07	*r* = −0.05, *p* = 0.48
**LPT**		*r* = −0.13, *p* = 0.07	*r* = 0.12, *p* = 0.09	*r* = 0.06, *p* = 0.37
**RDT**			***r* = −0.16, *p* = 0.02**	***r* = −0.32, *p* = 0.00**
**SLJT**				*r* = 0.12, *p* = 0.07

## Data Availability

Data will be made available in an online public depository.

## Appendix A

**Table A1:** Skewness and kurtosis values of all the measures included in the study.

	HP		HN		LP		LN	
	Skewness	Kurtosis	Skewness	Kurtosis	Skewness	Kurtosis	Skewness	Kurtosis
**Anxiety ^a^**	0.83	2.26	0.63	−0.75	0.27	−0.66	1.18	1.27
**Good/Bad_B ^b^**	−0.08	−0.33	−0.30	0.08	−0.84	1.59	−0.37	−0.87
**Awake/Tired_B ^b^**	0.56	−0.48	0.08	0.18	0.48	−0.38	0.08	−0.89
**Calm/Nervous_B ^b^**	−0.14	0.60	−0.72	0.00	−0.60	−0.06	−1.28	3.61
**Orientation ^c^**	−1.16	1.10	−0.53	−0.88	−1.24	2.02	−0.57	−0.21
**Adjusted R2 ^d^**	−0.69	−0.58	−0.54	−1.30	−1.47	1.23	0.03	−1.57
**Metric Error ^e^**	0.05	−0.27	−0.12	−0.63	0.54	−0.59	0.12	−0.43
**Spatial Length Judgment Error ^f^**	0.28	−0.95	1.60	2.14	2.02	5.01	0.85	−0.50
**Temporal Length Judgment Error ^g^**	0.66	−0.04	0.36	−0.77	1.51	1.71	1.42	1.73
**Maps ^h^**	−0.79	−1.04	−0.48	−1.23	0.27	−1.53	0.50	−1.13
**Good/Bad_A ^i^**	0.11	−1.02	0.14	0.60	−0.42	0.00	−0.24	−0.71
**Awake/Tired_A ^i^**	−0.05	−0.92	0.60	−0.17	−0.13	−0.01	0.07	−0.55
**Calm/Nervous_A ^i^**	−0.10	−0.28	−0.68	−0.18	−0.22	−0.69	−0.38	−0.62
**MD_Good/Bad ^l^**	−0.02	0.62	−0.05	0.70	−0.92	0.15	−0.69	0.40
**MD_Awake/Tired ^l^**	0.68	2.02	0.49	0.13	−1.66	2.42	−0.01	−0.37
**MD_Calm/Nerv ^l^**	−0.33	−0.07	−0.77	0.02	−0.84	2.66	−0.08	0.04
**Recognition ^m^**	−1	−0.07	−0.98	0.10	−1.83	3.55	−0.84	−0.40

^a^ anxiety level measured before the experiment; ^b^ dimensions of the mood questionnaire measured before the experiment; ^c^ orientation abilities measured before the experiment; ^d^ measure used for the mental walking task; ^e^ metric error used during the absolute position landmark task; ^f^ spatial length judgment (errors in meters); ^g^ temporal length judgment (errors in seconds); ^h^ map accuracy (route drawing task); ^i^ dimensions of the mood questionnaire measured after the experiment; ^l^ mood change between before and after the experiment; ^m^ measure used for the recognition task.

## Appendix B

Semantic relationship between landmarks in the four experimental conditions.

(1) Latent semantic analysis (LSA: http://lsa.colorado.edu/ accessed on 18 June 2021). This analysis allowed the semantic relationship between the elements/items depicted by images for each experimental condition to be quantified at a more conceptual level. The words introduced for the computations were as follows:
  - clouds, flower, courtyard, field, fork, wire cutters, plate (LP condition);
  - dog, snake, spider, gun, fork, wire cutters, plate (HN condition);
  - garbage, exhaust gas, cemetery, shredded paper, fork, wire cutters, plate (LN condition);
  - baby, puppies, kiss, fireworks, fork, wire cutters, plate (HP condition).

In total, twenty-one combinations (i.e., two entries at a time from a set of seven entries) were assessed for each experimental condition and the corresponding value was calculated. Each value indicated the level of semantic relationship between two images (from 0 to 1). These values were then entered in a Kruskal‒Wallis ANOVA with the experimental condition (4 levels) as independent variable. Results showed no significant difference between the conditions (H(3) = 0.38, *p* = 0.94) (HP: M = 0.10, SD = 0.09; LP: M = 0.08, SD = 0.09; HN M = 0.10, SD = 0.11; LN M = 0.09, SD = 0.07). This indicates that the experimental conditions did not differ in the strength of the semantic relationship between the images located along the route.

(2) Semantic similarity index (ISS). This index was calculated by using the images from all the four experimental conditions. To this aim, we presented to 20 participants (all university students, 10 females, age range: 20–25) all the 16 emotional images at the same time and asked them to divide them in four groups. Crucially, participants were free to decide the grouping criterion. Afterwards, the ISS between the images was calculated for each group of images indicated by the participants. The score was based on the number of times participants spontaneously put together the images of the same experimental condition (e.g., if the participant spontaneously grouped together all four images of the HP condition, he/she was given a score of 1 on that condition, if he/she put only 3 images together, he/she was given a score of 0.75, if only two, a score of 0.5, otherwise 0). Therefore, each participant received four scores, one for each experimental condition. A one-way ANOVA was carried out on the semantic similarity scores by using the experimental condition (4 levels) as independent variable. Results showed that, also in this case, the strength of the semantic similarity between the images did not differ between the four experimental conditions (H(3) = 4.76, *p* = 0.19) (HP M = 0.69, SD = 0.18; LP M = 0.72, SD = 0.32; HN M = 0.76, SD = 0.17; LN M = 0.75, SD = 0.18).
